# Oxygen Gas a Remedy in Disease

**Published:** 1876-06

**Authors:** G. B. Heard

**Affiliations:** LaGrange, Ga.


### OXYGEN GAS A REMEDY IN DISEASE.

By G. B. HEARD, M.D. of LaGrange, Ga.

Oxygen as a distinct element was discovered about one hundred years ago, by Dr. Priestly, and its therapeutical history began almost with the period of the discovery. He learned that it constituted that portion of the atmosphere by which animal life is sustained. By confining a mouse in a jar containing a limited quantity of this gas, he found that it lived twice as long as when confined in the same amount of common air. This fact engendered in his mind the belief that where there was a deficiency of vitality in man, caused by disease, it could be fully restored by this gas. But he had misgivings that it might excite too great an action in the system, "causing the patient to live too fast;" an idea which prevails even to the present day in the minds of professional men who have not taken the time and pains to investigate it. By reason of the great perfection of chemical apparatus, this erroneous idea has been dispelled from the minds of those who have fully investigated this subject. It is no more a stimulus to the system than food is a stimulus to the stomach, sound to the ear, or light to the eye. It is nature's own stimulant. Notwithstanding the remedial virtues were thus early foreseen, it was also accompanied with this erroneous idea, which took firm root in the professional mind, preventing much good which might otherwise have been attained.

The attention of scientists, for fifteen or twenty years after the discovery of oxygen, was directed mostly to its physiological and chemical relations to animal life. Lavoisier demonstrated the composition of the atmosphere and the effect produced in the blood by respiration. By this time the physicians of England, Germany, and France had become very much interested in investigations of this element, and their researches led to the fact that a never-ceasing supply of oxygen was necessary to sustain animal life; that food and drink, and even the nitrogen of the atmosphere, might be withheld for hours without detriment to life; but the moment oxygen is withdrawn, that moment the animal begins to die.

Its first application in therapeutics, so far as I can find, was in 1783, by Calliens. The following year it was administered to a young lady by a physician of Geneva, and with great benefit. About this time the French government desired that the members of the Academy should make a fuller investigation of the "vital air," and Fourcroy was selected to prepare a report, but he went off into a speculative channel, and nothing of practical benefit was attained. Indeed, his efforts did more to retard the real virtues of the remedy, by reason of his application of it with relation to the agent employed.

It appears that the credit of placing oxygen gas on a practical footing as a remedy belongs to Beddoes. He unfortunately seems to have accepted Fourcroy's report to the French Academy as truth, that the therapeutical effects were in accordance with the relation of the agent employed to the oxygen of the system; that in phthisis there was already an excess of oxygen present, and therefore the administration of it in that disease would ultimately accelerate the inflammatory process. Beddoes, accepting this as a truth, failed to try it in that disease, which accounts for its absence in the following table, taken from the British Library. He, with Sir H. Davy and James Watt, had a building erected with suitable rooms, and these apartments being charged with oxygen gas, in which the patients were allowed to pass a certain time daily. The table below is given as the results, and they are perfectly wonderful when we take into consideration the imperfect chemistry of that time, and their limited knowledge of relieving the gas of all irritating impurities. It has been truly said that a more brilliant triumvirate was never brought together in the furtherance of a scientific object.

| CASES TREATED. | Cured. | Relieved. | Not Benefited. |
|---|---|---|---|
| Obstinate Ulcers | 2 | 2 | ............ |
| Leprosy | 5 | ........ | ............ |
| Spasms | 3 | 2 | ............ |
| Gutta Serena | ...... | 2 | 3 |
| Chlorosis | 5 | 2 | ............ |
| Epilepsy | 1 | ........ | 5 |
| Asthma | 10 | 9 | 3 |
| Cancer | ...... | 3 | ............ |
| Dropsy of Chest | 2 | 1 | 1 |
| Hypochondria | ...... | 1 | ............ |
| Dyspepsia | 3 | 1 | ............ |
| Dropsy | 2 | 1 | 1 |
| Hydrocephalus | ...... | 1 | ............ |
| Headache | 2 | 2 | ............ |
| Poison by Opium | 1 | | ............ |
| Paralysis | 2 | 1 | 1 |
| Scrofulous Tumors | 2 | ........ | ............ |
| Deafness | 1 | ........ | ............ |
| White Swelling | 1 | ........ | ............ |
| Scorbutus | 1 | ........ | ............ |
| Venereal | 1 | ........ | ............ |
| Melancholy | 1 | 1 | ............ |
| General Debility | 1 | ........ | ............ |
| Continued Fever | 1 | ........ | ............ |
| Intermittent Fever | 1 | ........ | ............ |
| Coldness of Extremities | 1 | ........ | ............ |

"In relation to the number and variety of cases in which it was used and found beneficial, may suggest at first sight a certain air of charlatanism, but subsequent observers have corroborated nearly all his statements. Nor when we consider the physiological relations of oxygen is it more surprising that its use should be applicable to a larger range of cases than that modification of diet should be beneficial in so many divers diseases."—*Essay by Dr. Smith.*

Other great physicians were engaged about this time in making observations and experiments in diseases with oxygen. Hill and Thornton, in Great Britain, and Gertanner and Odier of Geneva; but owing to the impractability of chemical manipulations, it was allowed to fall into disuse. Dr. Hill's work on the Medical Uses of Oxygen, published about this time, gives by far the greatest number of cases thus treated, but his observations were made before the days of physical exploration of the chest, and due allowance must be made in his report for possible error in diagnosis. In one of the early volumes of *Sillman's Journal* there are recorded some interesting cases of phthisis cure by the persevering inhalations of oxygen gas. Chaptal records a similar case, with like results, in his work on chemistry.

Notwithstanding these brilliant and satisfactory results, it was allowed to die out with its original promoters, caused, doubtless, by the difficulties of chemical manipulation and want of proper chemical apparatus. The subject of oxygen as a remedial agent, after having lapsed so often, was revived by Dr. S. B. Birch, of London, who wrote a volume on it about 1868, in which he claims to have presided at the renaissance, and also claims to have done everything towards placing it on a sure footing. In my humble opinion he deserves great credit for his efforts to place so valuable a remedy before the profession. Demorquay, of Paris, has issued a large work on inhalation, in which he records a great number of cases treated with oxygen—some with success and others without benefit. His work is said to be the most candid of all works written on this subject. Dr. Herman Beigel also has issued a work on inhalation, in which he records cases treated with oxygen with more or less success. He invented an apparatus for the production of the gas according to Fleetmann's process, by the decomposition of chloride of lime by cobalt.

In the past few years a new apparatus has been invented, by which the oxygen is taken from the atmosphere in large quantities, by exposing manganate of soda to a very high heat in iron retorts, and while in that heat a current of air is passed over it. This results in the absorption by the salt of a large portion of the oxygen. The current is then cut off and superheated steam substituted. The steam withdraws the oxygen from the salt and carries it into a condenser, from which the oxygen passes into a gasometer in a pure state. This apparatus is the invention of Tessie du Motay, and one of them is in operation in the city of New York, which places this valuable remedy within the reach of every professional man, not only of the city, but to those of the contiguous cities and towns.

Recently quite a number of processes have been added to the list of obtaining oxygen with greater or less facility, but the only process by which physicians of interior towns and cities can obtain it pure—which is of the very highest importance—is by the combustion of chlorate of potash. An apparatus consisting of a brass retort, in which the chlorate of potash is placed, is fastened to a head piece, which is continuous, to the bottom of a wash jar, half filled with an alkaline mixture, and this jar is connected to a second wash jar, with a like quantity of the alkaline mixture, into which the gas passes from the wash jar No. 1, and re-washed in No. 2; from thence it is passed into a rubber bag, from which it may be inhaled freed from all irritating substance. The quantity given will vary in individual cases, from two gallons daily to twenty or even thirty gallons in urgent cases of dyspnœa. Even eighty gallons have been inhaled in a day without any bad effects. The inhalations are generally given morning and evening. The mode of administering is simple. The flexible tube of the gas bag terminates in a mouth-piece, which is inserted into one nostril, and the other nostril remains free. At the moment of inspiration the flexible tube (which is held tightly between the thumb and index finger) is relieved of the compression, and the gas allowed to escape through the tube into the lungs; and during expiration the tube is again compressed, to prevent the products of respiration being thrown into the bag with the oxygen. By this means any quantity of air desired may be given with it.

*Physiological action of Oxygen.*—On this point Dr. A. H. Smith says: "In regard to the physiological action of oxygen, the first question to be determined is, whether it is possible to cause the blood to take up more oxygen than it receives from the atmospheric air: whether the point of saturation is not attained in ordinary respiration. On this point there was formerly but one opinion. It was thought that there was practically no limit to the power of the blood to absorb oxygen. This idea was no doubt based upon the known energy of combustion in pure oxygen gas, and the supposed identity of that process with the retrograde metamorphosis of animal tissue. It was held that the inhalation of pure oxygen would induce rapid chemical action within the body; that a state of general inflammation would ensue, and that destructive metamorphosis would be so much more active than the process of reconstruction, that the vital machinery would soon be spoiled and rendered incapable of continuing its action. But after a time it was observed that these extreme results did not actually take place; that an animal could remain for a number of hours in pure oxygen without sustaining any apparent injury. This led certain observers to the conclusion that the blood corpuscles became saturated with oxygen when common air was breathed, and that it would take up no more, no matter how much was presented to it in the air-cells of the lungs. This view was defended by Regnault and Reiset, who endeavored to sustain it by the following experiment: They confined animals in oxygen, and after a time examined the gas, and found that it contained no more carbonic acid than would have been exhaled in the same time if the animals had respired atmospheric air. That these experiments were not conclusive will become apparent as we proceed.

"Experiment 1.—A rat was confined in a jar containing about a gallon of pure oxygen, and inverted over water. At the end of two and a half hours death took place. On opening the body all the internal organs were found to be of a bright red color.

"It will be observed that no provision was made for removing the carbonic acid and other products of respiration, and that the gas must have become very impure. In the following experiment this omission was corrected:

"Experiment 2.—A pigeon and two mice were confined respectively three, four, and five hours in jars of oxygen, having a strong solution of caustic potash in the bottom, under a stage upon which the animal rested. The jar was so arranged at the same time that a small stream of oxygen from a rubber bag was constantly flowing into it, and escaping by an aperture of like size. The solution of potash absorbed the carbonic acid, while the gradual change of atmosphere in the jar was sufficient to prevent a sensible accumulation of other impurities. These animals, when killed, did not present any appreciable change.

"The conclusion which I have drawn from these experiments is that the lively red color of the lungs, heart, liver, etc., which are described and which I have myself seen, does not depend upon hyper-oxygenation alone, but also on a coincident retention of carbonic acid in the tissues. My view is, then, that if pure oxygen be taken into the lungs, only as much will be absorbed by the blood as would be taken up from the air under circumstances involving the greatest possible physiological demand for oxygen.—*Smith's Essay.*"

The blood cannot absorb more oxygen than the greatest demands of nature require, and this point is a complete saturation, which is quickly attained by inhalation of pure oxygen, and this being purely a physiological action, does not bring about any morbid action in the system.

How does oxygen act as a curative agent? In ordinary respiration we take in a certain amount of atmospheric air, which contains one-fifth of oxygen, and we immediately exhale four-fifths of what was inhaled, that is, the nitrogen and carbonic acid gas, the oxygen having formed a chemical union with the carbon, producing that gas; the blood thereby being relieved of impurities is then sent on its health-dispensing round through the system. In diseases of the respiratory organs, the breathing capacity being lessened, there is always a deficient amount of oxygen taken from the ordinary medium; therefore the blood cannot be entirely relieved from impurities, and hence the partially-relieved blood is sent again and again on its rounds, and these clog the channels of the circulation. Now what is more reasonable than to supply, by air much richer in oxygen, this deficiency, to act as a solvent to these accumulations?

The application of oxygen as a remedy naturally falls into that class of diseases in which there is defective nutrition, and those which give rise to dyspnœa. Asthma being of that class in which the dyspnœa is so great, it is not strange that it should have been one of the first diseases in which oxygen was applied. Every action of the patient seems to be a plea for more air. From a view of the table given above, it will be seen that Beddoes employed it in twenty-three cases. Demarquay reports two cases—one positively cured, and the other greatly relieved. Birch reports a case of absolute cure. Dr. H. Beigel gives a report of three cases, two of them hereditary, in which oxygen was used with inhalations of liq. potas. arsen.

The paroxysms became less and less severe, and finally ceased altogether.

Dr. A. H. Smith, U. S. A., reports on the case of ex-Secretary Stanton, in whose case, during his last illness, asthma was a serious complication. He says: "In a conversation with his medical attendant, Surgeon General Barnes, who procured from New York oxygen in a compressed state, of which he gave him daily three to four cubic feet. It controlled the paroxysms completely, and, so far as the asthma was concerned, met every indication. In a recent conversation with me, General Barnes expressed himself warmly in favor of the gas in similar cases, and stated that both he and his illustrious patient were entirely satisfied with its effects."

I have given it during a paroxysm with the effect of relieving it instantly. Dr. Beigel, in his work on inhalation, reports a severe case of croup in which the usual mode of treatment had failed; the respiration being forty per minute and noisy; the pulse too frequent to count; lips livid; face pale and agitated by convulsive movements. The inhalation of one cubic foot of oxygen was followed by decided improvement, and in the course of two or three hours the child fell asleep, awakening convalescent and making a prompt recovery.

There are numbers of similar cases on record, in which the use of oxygen inhalations were followed by the same results, and I have no doubt in my mind that its timely use will do all that tracheotomy can do in supplying the blood with oxygen and giving *vis medicatrix naoturæ* an opportunity to throw off the adventitious matters. If it should be withheld until there is mechanical engorgement, and a consequent poisoning of the nerve centres, of course it can do no good.

This remedy is now being used extensively in the treatment of pneumonias, dyphtheria, scarlet and typhoid fevers.

Dr. Golden reports in the *Lancet*, March 10, 1866, a case of double pneumonia, with great dyspnœa, which was resolved in four days by the use of oxygen. I have had only a single opportunity of giving it a trial in double pneumonia; the patient being too far gone the results were nothing. In chronic bronchitis the effects of this remedy are observable in a very short time. I have often seen the good effects in this disease in two or three days.

*Capillary Bronchitis.*—"In this affection" (I quote Dr. Smith) "oxygen is of great value, not only in relieving dyspnœa, but also in diminishing the formation of mucus on the lungs, which latter is in a great measure the mechanical result of excessive inspiratory effort. For whenever the inspiratory effort is in excess of the capacity of the air passages to supply the necessary air, the atmospheric pressure within the chest is necessarily diminished, and turgescence of the blood-vessels of the lungs follows as certainly as hyperæmia of the skin follows the application of a cupping-glass. Inhalations of oxygen, by satisfying the *besoin de respirer*, removes the necessity for employment of excessive inspiratory force, and in this way lessens the congestion of the mucous membrane and diminishes the tendency to effusion. Hence it is not only palliative, but curative.

During my stay in Jacksonville, Florida, this winter, my friend and partner, Dr. J. D. Mitchell, was requested to see a lady who was suffering from excessive dyspnœa, the result of infiltration, caused by a hemorrhage from the lungs two or three days previous. He found her with considerable congestion of the lung and the inspiratory effort attended with excruciating pain; also alarming delirium. He administered to her continuously for about one hour oxygen gas, when she was entirely relieved from all pain and became perfectly conscious. Being so much pleased, she desired taking it for a longer time.

*Phthisis.*—This disease will now be considered; the history and symptoms of which prove it to be one in which there is defective or impaired nutrition and faulty respiration, and in which local lesions are a manifestation of it. The effects of oxygen upon ulcers, applied either locally or by inhalation, is very remarkable. Beddoes, in the early days of oxygen, made observations of the effects of it in ulcerated surfaces and ill-conditioned ulcers. Ulcers of years standing, which had resisted all other topical and constitutional treatment, were healed by the use of oxygen in a short time. I administered it to a gentleman last September, who was suffering from an anal abscess, the result of chronic diarrhœa and great debility. In three weeks this abscess of two or three years standing was entirely cured. Demarquay cites several cases of ulcers of great extent cured in a very short time from the use of oxygen. A great number of cases can be cited which go to prove the wonderful power of it in inducing cicatrization. Dr. Birch attributes this wonderful effect to its purifying powers, and says that no other remedy will compare with oxygen as, in common parlance, a blood purifier. This wonderful effect and power over ulcerated surfaces being a well-established fact, why should it not produce the same effect on ulcers of the lungs, when they are exposed not only to the influence of direct contact, but also partake of its effects acting through the circulation.

A change of climate, where the patient can take exercise for the greater part of the day in the open air, is the stereotype prescription in all cases of incipient phthisis. The object in view is two-fold: first, by exercise in the open air the blood is more quickly oxidized, and the patient's zest for food is enhanced; secondly, the food in the stomach is better prepared for the formation of new tissue, by reason of the greater amount of oxygen taken from the atmosphere by out-door exercise. "In diseases involving defective nutrition" (I quote from Dr. Smith's Essay), "the benefit derived from the use of oxygen in cases not primarily involving respiration, is to be explained on the principle that it aids defective nutrition. The replacement of old and effete matter by that which is new and active is fully as much the work of respiration as digestion. Without the oxygen derived from respiration, tissue change would be immediately checked. If the blood did not convey to the tissues the requisite supply of oxygen, the gastro-intestinal might do its part, and the food might be absorbed, but there the process would be arrested. The material would be on the spot, but the structure would not be repaired. Nor is it enough to present the usual supply of oxygen to the blood in the lungs. The blood itself must be in a proper condition to receive it, or it cannot reach the tissue. The quality of the blood by which it takes up oxygen depends upon the exactness of its chemical constitution. A slight variation in this will affect its absorbing power. But a condition of the blood which prevents the absorption of sufficient oxygen from the diluted medium usually respired, may allow its absorption from a medium in which a greater proportion of oxygen is contained. A deficient absorbing power may be supplemented by an increased supply of the material to be absorbed. It is on this principle that even small quantities of artificial oxygen inhaled each day are capable of producing such decided results in cases appropriate for its use."

"But there is one form of disease which at the same time deprives the blood and interferes with the functions of the lungs, thus striking a double blow at the functions of hæmatosis. It may therefore be appropriately considered—first, as intermediate between those cases in which respiration is principally involved, and those in which nutrition is chiefly at fault."

"Among chronic diseases pulmonary phthisis was the one which offered the most tempting field for the use of oxygen. It was natural to expect that the profound dyscrasia which lay at the root of the disease might be favorably modified by an agent bearing such intimate physiological relations to the normal blood. At the same time there was room to look for a double local action within the lungs. What might be the result of bringing an excess of free oxygen into direct relation with the tubercular matter in the pulmonary tissue was a question not less interesting than what might result from the local action of the gas upon the ulcerated surfaces with which it would come into contact."

With regard to the effect of oxygen upon the system generally in phthisis, we have abundant evidence to show that it, as a rule, in this disease as in others of defective nutrition, favors digestion and promotes assimilation, and results in a gain in weight, as will be seen hereafter; and by reason of its solvent properties eliminates all carbonaceous matter from the blood, and thereby sending pure blood to the different portions of the system. We are taught that oxidization is the first step in the decay and disintegration of dead organic matter, and therefore, by bringing pure oxygen into contact with deposits in the lung, it will certainly bring about that metamorphosis, thereby promoting its absorption.

To sum up the effects of oxygen in this disease: it has the power, in a marked degree, of controlling the circulation in the evening fevers; the cough begins to lessen gradually, and generally, after the daily use of it for four or six weeks, the cough is entirely relieved. When there is profuse expectoration, it too begins gradually to diminish *pro tanto* with the cough, and in chronic bronchitis it is notably the case. The pains in the chest rapidly disappear under its use. Night sweats are by some authors traced to the escape of the hydrocarbons by the skin from the blood, and in phthisis the fatty matter in the blood appears to act as a stimulus to the sweat glands. Now if the quantity of oxygen be increased artificially in the system, oxidization of the fats will take place, and the sweating will cease, which always takes place in a very few days under oxygen inhalations. It has already been stated that oxygen applied locally to a wound or ulcer stimulates it and promotes granulation and cicatrization; so will it act carefully applied in ulcers or cavities of the lung. Of course, the earlier in this disease resort is had to oxygen the more confident we are of success.

Dr. Birch says, by the use of oxygen the cure of consumption in its earlier stages should be the rule rather than the exception. Dr. Smith gives it as his opinion that the treatment of phthisis by oxygen inhalations is by far better than any and all other remedies combined. I believe that a very large per cent can be cured by the judicious use of oxygen alone. The addition of cod-liver oil to the treatment, in appropriate cases, is beneficial, as the oxygen aids the digestion of this substance.

I will now give the result of the oxygen treatment in the hands of different physicians, by recording a few cases:

Case I. Reported by Demarquay, of Paris.—X., age twenty-three; tubercles in both lungs; cavity in left size of an egg; greatly emaciated, pale, anæmic, profuse expectoration, intense fever in afternoon, diarrhœa.

March 1st. To take four litres of oxygen in ten of air daily. 3d. The cough is less frequent, expectoration less abundant; slept well. Increase to twelve litres (four gallons). 4th. A little appetite, but little cough, no expectoration. 6th. Great appetite, sleeps well, physiognomy better. 8th. Increase to fifteen litres. 10th. Patient has been up and about for the last two days; appetite so great that after eating the dinner provided by the institution, he goes out and dines again in the city. Face has more color, cheeks filling out, respiration easier. 16th. Cavity still remains, but the surrounding tissue which was hepatized, now performs its function. 19th. Able to take a long walk. 26th. Cough and expectoration have entirely disappeared; no gurgling as formerly; respiration still amphoric. April 30th. Discharged in a very satisfactory condition.

Case II.—Madame DeB., aged twenty-seven; tubercles in both lungs; emaciation, frequent cough, profuse expectoration, almost no appetite, abundant night sweats. The appetite improved and the strength increased, and on the twenty-seventh day of treatment by oxygen alone, patient was able to give a dinner party, and preside for two hours at the table. The menses, which had been absent for five months, returned. The cough and expectoration, though less, persisted during the summer, and toward autumn, the use of the gas having been for some time suspended, a relapse took place, and death followed the ensuing February.

Case III. Reported by M. Monod (quoted by Demarquay). M. C. B., age twenty-seven; for some years has had very abundant hemorrhages; signs indicated a number of cavities and an abundant deposit of tubercles, especially in right lung. At the commencement of treatment was so feeble that he could only be moved from bed to sofa. Abundant muco-purulent expectoration, complete anorexia. Twelve litres of oxygen were given twice a day, and within a brief period he had so far improved that he was able to walk in the garden, and even to attend occasionally to business. For sixteen months the treatment has been continued, during which time there has been no return of hemorrhage; the expectoration is now insignificant and the cough infrequent. The appetite is habitually good; still the disease is making progress; the pulse is frequent and the strength is less than last year. But the oxygen has restrained the march of the disease, which last spring seemed to have arrived at the last stage. The improvement followed so immediately upon the administration of the gas, that it could not be attributed to any other cause.

Birch relates two cases, in one of which auscultation, percussion, and microscopical examination of the expectoration, confirmed the diagnosis as regards tubercular consolidation and central cavity. From the very first dose of oxygen, a diminution in the sensations of irritation and weakness of the chest could be felt by the patient. Within a month marked improvement evidenced itself, both in the lung and general health, and at the termination of four or five months steady treatment the flattening of the chest had given away to almost perfect symmetry. Two years after the commencement of the oxygen, she was quite well, married, and has ever since enjoyed good health.

The second case he reports the upper third of the right lung as being full of small cavities, which, breaking into one, under the use of oxygen contracted, causing considerable depression of the walls of the chest. Treatment was continued for a year. Patient quite well six years afterwards.

In a patient of Dr. Frauenstein, of New York city, there was dulness at apex of right lung, with tubular breathing and moist rales, and accompanied by the rational symptoms of incipient phthisis. No other treatment was employed than the daily inhalations of from eight to twelve gallons of oxygen. In four weeks the abnormal auscultatory sounds had disappeared, and the dulness was scarcely perceptible. The subjective symptoms, such as dyspnœa on ascending stairs, etc., had also disappeared, and the patient had gained flesh.

Case reported by Dr. A. Smith.—Mrs. S., age twenty-eight, was sent to me by a physician, with the request that I would try the effects of inhalation of oxygen. She had had a distressing cough for three months; expectoration profuse, slight dulness and tubular breathing at summit of both lungs; extremely pale and anæmic; utter disgust for food; menstruation had been growing more and more scanty for some months past, and the last two periods had failed entirely. Three weeks before, began the use of iron, but had to abandon it, as it disagreed with stomach and caused headache. Six hundred cubic inches of oxygen were inhaled every morning, mixed with about four times its bulk of air. For the first week little if any benefit was experienced; after that the appetite began to improve. On the tenth day of treatment the menses returned, and were more abundant and of a better color than for many months previous. The appetite now became very great, the patient declaring that when returning from office she could scarcely wait to reach home, so great was the desire for food. Iron was now borne without difficulty, but after two or three weeks she found that she could not take iron and oxygen the same day without headache, but she could bear either separately. At this time the cough improved rapidly. A simple expectorant mixture was ordered, but was taken very irregularly. In six weeks from the beginning of the treatment the cough had ceased entirely; the patient had gained flesh and strength, and considered herself quite well. Two months later she called at my office, and reported that since I last saw her she had gained seven pounds; the menstruation had continued regular, and that, in short, she had never been better in her life. Respiration normal at summit of both lungs.

Case by same.—On 7th December I administered oxygen to Miss H. A., patient of Dr. Frauenstein, of New York, age twenty-five. She was then extremely emaciated, had distressing cough, and presented all the symptoms of pulmonary phthisis. After the first visit she continued the use of the gas under the direction of Dr. F. The 3d of January I received a note from the Doctor, stating that the area of dulness in the lung was becoming less, and that although the cough continued, she was gaining flesh. He wrote: "She coughs and grows fat." Three days later she called at my office. The change in her appearance was marvellous. Her previously hollow cheeks had become round and full, and her whole person seemed to have expanded. It was like the change one sees after the recovery from continued fever. Certainly, up to this time, nothing could be more gratifying than the progress of this case.

To more fully test the merits of this remedy in diseases of the lungs, I visited Jacksonville, Florida, last December, the great winter resort for consumptives. There I met Dr. J. D. Mitchell, a physician of high repute and long a resident of the place. In him I found an earnest coadjutor in promoting the interest of science. We treated by oxygen gas inhalations quite a number of patients; a history of some of the most important I append below:

Case I.—Mr. R. Millnor, age thirty, had frequent hemorrhages for the past four years. On 6th December, 1875, bled to the amount of three pints. 8th. Began treatment. Auscultatory examination revealed consolidation of the right lung and some protrudence of the anterior right side. No air admitted in the right lung. Had had, about two years previous, a large abscess, which discharged pus for eight months. This abscess was situated in front of the lower portion of the right lung, and had caused extensive adhesion at that point. He continued to spit blood up to the 9th. He was then taking the following as a hæmostatic:

℞.—Ergotine, . . . . . . . grs. v.
Extract Lactucarium, . . . . ℨj.

M. Div. into pills No. xx. S. Take two pills every four hours.

December 15th. No more spitting of blood; cough and some expectoration. At the time he began treatment there was a large scrofulous tumor, which had gradually made its appearance just below the elbow. He continued the oxygen treatment for eight weeks, at which time his lung was freely admitting air, and presented all the normal evidence of performing its functions, except over the cicatrix where the abscess had discharged two years previously, and the scrofulous tumor of the arm had been entirely removed. His case was remarkable; he telling me that he had often had as many as three hemorrhages a day for three weeks consecutively, and had never before had a hemorrhage without a succession of them. He never had a symptom of hemorrhage after the second day of treatment. He had lost twenty-four pounds in weight the month previous to this treatment. The wasting was immediately checked, and at the termination of eight weeks was steadily gaining flesh. The progress of his case was altogether satisfactory.

Case II.—Wm. Fahey, Bellville, C. W., age twenty-three, brick-mason; had been sick eighteen months. Dec. 16. Had not slept for four nights; very much weakened; great pain in right lung; fever cough very frequent, and profuse expectoration, with abundant night sweats; broncho-consumption; considerable gurgling in right lung. 17th. Slept some after the evening treatment. 18th. Directed to take cod-liver oil and hypophosphite of lime three times daily. 19th. Slept well; appetite improving; less cough; same abatement in fever; pulse 90, and respiration, previous to treatment, was 50 per minute, now 30; evident improvement in all symptoms. 27th. No fever; coughs but little, and no night sweats; gaining strength; can take a walk of some considerable distance without fatigue, whereas, previous to treatment, he could take but very few steps, on account of dyspnœa. He continued to improve in every particular, and, after five weeks treatment, desired to resume his trade, which he had been compelled to abandon for eighteen months. After consulting me on the subject, I gave my consent to his resuming work, which he did, and previous to my departure from that place had been regularly at work daily for two weeks without any detriment, and as I often visited the building on which he was at work, I could not help but think that he was a living monument to the virtues of oxygen. He had been examined by a leading physician of the city, and was told that his case was hopeless; and this examination took place only a few days before he began the oxygen treatment. I received a letter from him a short time ago, and he writes that he is as well as he ever was. It is proper to state that he gained sixteen pounds in the five weeks treatment. He continued the treatment under Dr. Mitchell's supervision after I left for two weeks.

Case III.—B. F. Schenck, Vevay, Indiana; age forty-two; sick for two years; had several hemorrhages; cough and profuse expectoration; fever in afternoon, chills in morning, with cold extremities. Dec. 28. Began treatment; four gallons of oxygen twice daily, and quinine in the morning. January 5. Improvement in all symptoms; less cough and expectoration; appetite improving. Just one month after beginning treatment, he expressed himself as feeling quite well; no cough or expectoration, and was gaining flesh rapidly. His own language was, that "he was well before he knew it."

Case IV.—George Morner, Hamilton, Ohio; age twenty-five; tubercles at summit of both lungs; extremely emaciated; distressing cough; pale and very anæmic; very little strength; presented all the signs of pulmonary phthisis. Began treatment Dec. 26th. Jan 6. Less cough and expectoration. Jan. 13. Decided improvement in all the symptoms; his pale and hollow cheeks had become round, and were set off with a good color; was able to take a long walk. Continued the treatment up to Jan. 26th, when he felt well enough to give up treatment.

Case V.—Miss Nettie R., New York; age fifteen; had been sick just five months, and although I did not make any physical examination, her appearance indicated tubercular phthisis. She was pale and emaciated, with severe cough and profuse expectoration, and fever every afternoon, with night sweats; no appetite. Dec. 18. To take four gallons of oxygen, largely mixed with air, daily. 22d. Fever entirely gone, and less cough and expectoration; no night sweats; appetite improving. 25th. Still great improvement in all of the symptoms. The excellent effects of oxygen were very notable in this case. Just at this time I was compelled to visit my home, on account of a serious family affliction, and on my return, two weeks after, her father had left with her, and I lost sight of the case. No other treatment was given in this case, and of course the improvement was attributable to the oxygen.

We administered to a large number of other cases with great benefit; the cough and expectoration being lessened and appetite improved; but the time being of short duration, there were no definite results previous to my departure.

In other cases, there being no immediate benefit, the patients have become discouraged and abandoned the treatment without giving it a fair trial. In no case should a patient start with the treatment for a less period than four weeks, although it is frequently the case that notable effects are observed in a a very few days.

*Nasal Catarrh.*—In this disease I have never found any plan of treatment to compare with oxygen. It comes in direct contact with the nasal membrane, and relieves the thickened condition, and, where there is ulceration, causes it to heal in a very short time. The splendid effects in this disease were observed in the following case:

Miss D.; age twenty-five; suffering for eight years with nasal catarrh; complexion very sallow; constant feeling of constriction in the frontal region, with headache; careless in personal appearance; never going in society. After a two weeks treatment, the change in her appearance was so evident that those who were acquainted with her remarked it. At the end of six weeks she was dismissed, perfectly free from any symptom of the disease.

When there has been daily inhalation of oxygen for several days successively, there is a sense of freedom about the chest and an ability to expand it more freely than before taking the oxygen. It seems to equalize the circulation and overcome the coldness of the extremities, where that exists, without any change of the temperature of the body.

The foregoing is far from presenting a full view of the therapeutical results which have been attained by oxygen, but enough is shown to prove that it is a remedy that will fill indications and effect results in those diseases where the usual therapeutical agents are inadequate.

Below I append a letter from Dr. J. A. Baugh, of this city, he having used it in several cases:

MARCH 15, 1876.

*Dr. J. A. Baugh:*

DEAR SIR—Will you please give me your views in regard to oxygen inhalations as a remedy in pulmonary affections?

Respectfully yours,

G. B. HEARD, M.D.

LAGRANGE, GA., March 15, 1876.

*Dr. G. B. Heard:*

DEAR SIR—In reply to your question, I have to say that I unhesitatingly give my testimony in its favor. I am very familiar with Judge W. W. Turner's case, and have no hesitancy in saying that it restored him to a full and vigorous state of health, after suffering for four years with hemorrhage from lungs and with all the rational symptoms of phthisis. Dr. C. H. Lockhart, of this place, was for ten years a confirmed consumptive, and had hemorrhages from the lungs almost weekly. He had been compelled to abandon his practice for years. After two months treatment he was able to resume his business, and now regards himself as well, no symptom of the disease remaining. Other cases could be mentioned, but I deem it unnecessary. With my knowledge of the remedy, I am candid in saying that I think it destined to supersede all other treatment for lung affections in the near future.

Very respectfully yours,

J. A. BAUGH, M.D.

